# Identification of Genes and Metabolic Pathways Involved in Resin Yield in Masson Pine by Integrative Analysis of Transcriptome, Proteome and Biochemical Characteristics

**DOI:** 10.3390/ijms231911420

**Published:** 2022-09-28

**Authors:** Zhengchun Li, Luonan Shen, Qiandong Hou, Zijing Zhou, Lina Mei, Hong Zhao, Xiaopeng Wen

**Affiliations:** 1 Key Laboratory of Plant Resource Conservation and Germplasm Innovation in Mountainous Region (Ministry of Education), Institute of Agro-Bioengineering, Guizhou University, Guiyang 550025, China; 2 Institute for Forest Resources & Environment of Guizhou/College of Forestry, Guizhou University, Guiyang 550025, China

**Keywords:** masson pine, resin yield, transcriptome, proteome, biochemical characteristics, molecular regulatory mechanisms

## Abstract

Masson pine (*Pinus massoniana* L.) is one of the most important resin-producing tree species in southern China. However, the molecular regulatory mechanisms of resin yield are still unclear in masson pine. In this study, an integrated analysis of transcriptome, proteome, and biochemical characteristics from needles of masson pine with the high and common resin yield was investigated. The results showed that chlorophyll a (Chl a), chlorophyll b (Chl b), total chlorophyll (Chl C), carotenoids (Car), glucose (Glu), gibberellin A9 (GA9), gibberellin A15 (GA15), and gibberellin A53 (GA53) were significantly increased, whereas fructose (Fru), jasmonic acid (JA), jasmonoyl-L-isoleucine (JA-ILE), gibberellin A1 (GA1), gibberellin A3 (GA3), gibberellin A19 (GA19), and gibberellin A24 (GA24) were significantly decreased in the high resin yield in comparison with those in the common one. The integrated analysis of transcriptome and proteome showed that chlorophyll synthase (*chlG*), hexokinase (*HXK*), sucrose synthase (*SUS*), phosphoglycerate kinase (*PGK*), dihydrolipoamide dehydrogenase (*PDH*), dihydrolipoamide succinyltransferase (*DLST*), 12-oxophytodienoic acid reductase (*OPR*), and jasmonate O-methyltransferases (*JMT*) were consistent at the transcriptomic, proteomic, and biochemical levels. The pathways of carbohydrate metabolism, terpenoid biosynthesis, photosynthesis, and hormone biosynthesis may play crucial roles in the regulation of resin yield, and some key genes involved in these pathways may be candidates that influence the resin yield. These results provide insights into the molecular regulatory mechanisms of resin yield and also provide candidate genes that can be applied for the molecular-assisted selection and breeding of high resin-yielding masson pine.

## 1. Introduction

Conifers have evolved specific defensive traits and strategies to cope with herbivores and pathogens [1,2,3]. Oleoresin (hereafter ‘resin’) is a major defense of conifers, consisting of complex and variable mixtures of different monoterpenes, sesquiterpenes, and diterpenes [2,4,5,6]. These terpenoids from resin have a variety of applications in industry, including chemicals, pharmaceuticals, agrochemicals, food, and bioenergy [7,8]. From single cells or blisters to networked resin ducts, secretory structures of varying complexity are used to manufacture, store, and transport resin [1]. Resin ducts are abundantly present in the stem, roots, needles, and reproductive structures of many conifers [9,10].

In conifers, resin yield is a quantitative trait under moderate to strong genetic control [11,12,13]. Important genetic gains can be obtained by selecting high resin yielders [14]. The factors that influence resin yield are a series of genetic genes related to resin traits. The resin yield is mainly affected by photosynthesis-related genes (controlling photosynthesis-producing capacity) and resin biosynthesis-related genes (controlling resin-producing capacity) [15]. Previous studies demonstrated that resin yield was closely related to tree diameter, percentage of the live crown, photosynthesis, resin duct number, and resin duct volume [15,16,17,18]. The relationship between resin yield and resin components has been also investigated, and the results demonstrated that some terpenoids could be used as the diagnostic marker for high resin yielders [7,18,19]. To date, however, studies involved in the molecular regulatory mechanisms of resin yield have been very limited. Excavation of the genes involved in resin yield can facilitate the selection for high resin-yielding germplasm and shorten the breeding cycle in conifers.

As is well known, terpenoid biosynthesis is usually completed via methyl-erythritol 4-phosphate (MEP) and mevalonate (MVA) pathways. The MEP pathway begins with the condensation of pyruvate and d-glyceraldehyde 3-phosphate (G3P) to generate 1-deoxy-d-xylulose 5-phosphate (DXP), followed by intramolecular rearrangement and reduction. The MVA pathway includes the condensation of three molecules from the initial substrate acetyl-coenzyme A to MVA and other phosphorylation events [20]. It is thought that monoterpenes and diterpenes are biosynthesized by the MEP pathway, which are the major components of the conifer resin, while sesquiterpenes are biosynthesized by the MVA pathway which is less abundant in the resin [3]. In terpenoid biosynthesis, a set of enzyme genes, including geranyl pyrophosphate synthase (*GPPS*), farnesyl pyrophosphate synthase (*FPPS*), geranylgeranyl pyrophosphate synthase (*GGPPS*), monoterpene synthase (*MonoTPS*), sesquiterpene synthase (*SesquiTPS*) and diterpene synthase (*DiTPS*), are directly participated in the biosynthesis of monoterpenes, sesquiterpenes, and diterpenes [2,21]. The formation of diterpenoid resin acids (DRAs) involves further oxidation of diterpene synthase products by cytochrome P450-dependent monooxygenases of the CYP720B group [5,22]. The terpene synthases and CYP720B genes respond to real or simulated insect attacks by increasing transcript levels, protein abundance, and enzyme activity [6]. The exploration of key genes involved in resin terpene biosynthesis using multi-omics techniques plays an important role in revealing molecular regulatory mechanisms of resin yield.

With the development of next-generation sequencing technologies, advanced approaches have greatly contributed to biological studies in non-model plants. Pacific Biosciences Single Molecule Real-Time (SMRT) can capture a complete catalog of transcripts and their variants [23,24], whereas the technique has a high error rate, which can be corrected by the Illumina short reads (RNA-Seq) [25,26,27]. A combination of the second-and third-generation transcriptome can provide both full-length transcripts and the expression profiles of transcripts in specific samples, which is beneficial for excavating key genes and metabolic pathways involved in resin yield. The proteomic assay can provide more direct information on critical biological processes, such as terpenoid biosynthesis [28]. Iso-baric tags for relative and absolute quantitation (iTRAQ) is an extensively used quantitative technology in proteomic studies [29,30,31,32] that improves the accuracy and reliability of protein quantification in comparison with those of conventional 2D-polyacrylamide gel electrophoresis (PAGE) [33,34]. However, no studies had been performed concerning the expression changes of genes and proteins simultaneously, e.g., through the integrative analysis of transcriptome and proteome, and therefore less information had been available about the association between transcriptome and proteome profiles concerning resin yield in masson pine.

As one of the most important native tree species in southern China, masson pine (*Pinus massoniana* L.) plays a key role in providing wood and resin for industrial uses, and approximately 90% of resin is from this species. Resin yield is a significant economic trait and remarked genetic variations within different genotypes have been found in masson pine [14,35]. However, the molecular regulatory mechanisms of resin yield in masson pine are unclear. To better understand the molecular regulatory mechanisms of resin yield and to identify the key genes and pathways involved in resin yield at the transcriptomic and proteomic levels, in this study, Iso-Seq, RNA-Seq and iTRAQ were carried out in parallel to identify the differentially expressed genes (DEGs) and differentially expressed proteins (DEPs) in needles of masson pine with the high and common resin yield, followed by the functional analysis of these specifically or commonly regulated DEGs and DEPs. Subsequently, biochemical characterization was further combined to identify the key genes and pathways involved in resin yield. In the present research, the contents of photosynthetic pigment, sugar, and endogenous hormone were detected to determine the differences in biochemical levels between the high and common ones. Candidate genes and pathways related to resin yield were identified through RNA-Seq and iTRAQ. The results provide a theoretical foundation for elucidating the molecular regulatory mechanisms of resin yield and also provide candidate genes that can be applied for the molecular-assisted selection and breeding of high resin-yielding masson pine.

## 2. Results

### 2.1. Differences in Photosynthetic Pigment Contents between High and Common Resin Yield

**Figure 1:**
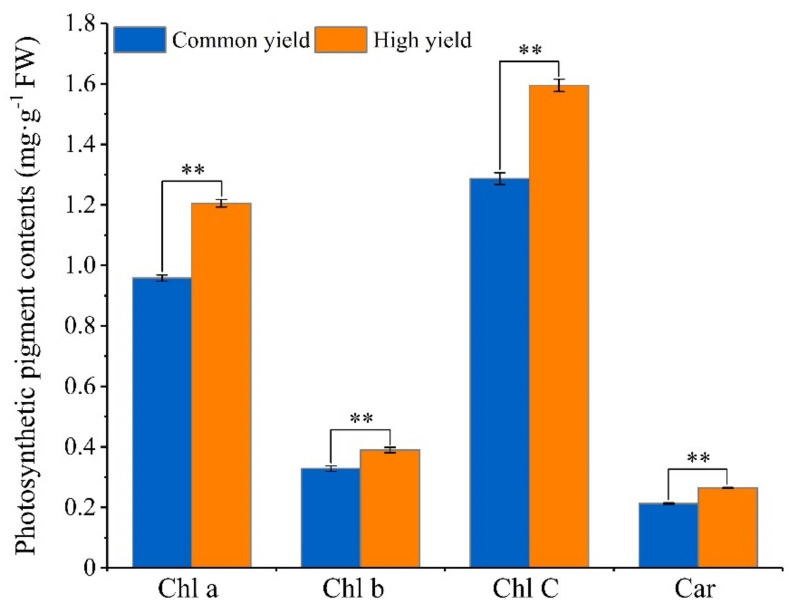
Photosynthetic pigment contents of masson pine needles in the high and common resin yield. FW means fresh weight. “Common yield” means the common resin-yielding clone, and “High yield” means the high resin-yielding clone. Error bars refer to the standard deviation (SD) of three biological replicates. ** indicates a significant difference between the high and common ones at *p* < 0.01. Chl a, chlorophyll a; Chl b, chlorophyll b; Chl C, total chlorophyll; Car, carotenoids.

To analyze the accumulation characteristics of the photosynthetic pigment between the high and common resin yield, we compared the contents of the pigment Chl a, Chl b, Chl C, and Car. The results showed significant differences in the photosynthetic pigment contents between the high and common resin yields. The contents of Chl a, Chl b, Chl C, and Car in the high resin yield were greatly higher than those in the common one (Figure 1).

### 2.2. Differences in Sugar Contents between High and Common Resin Yield

**Figure 2:**
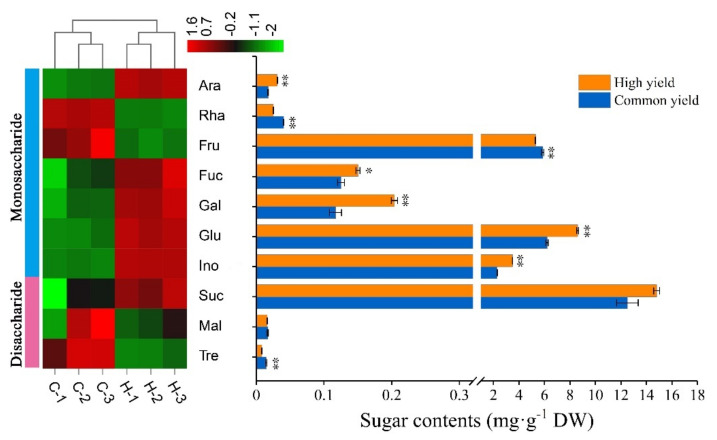
Sugar contents of masson pine needles in the high and common resin yield. DW means dry weight. “Common yield” means the common resin-yielding clone, and “High yield” means the high resin-yielding clone. Error bars refer to the standard deviation (SD) of three biological repeats. * and ** indicate significant differences between the high and common ones at *p* < 0.05 and *p* < 0.01, respectively. C-1, C-2, and C-3 represent three biological repeats of common ones, H-1, H-2, and H-3 represent three biological repeats of a high one. The color brightness represents the degree of the content difference, as shown in the color bar. Ara, D-arabinose; Rha, L-rhamnose; Fru, D-fructose; Fuc, L-fucose; Gal, D-galactose; Glu, glucose; Ino, inositol; Suc, sucrose; Mal, maltose; Tre, trehalose.

As shown in Figure 2, a total of 10 sugars were detected in the needles, including D-arabinose (Ara), L-rhamnose (Rha), D-fructose (Fru), L-fucose (Fuc), D-galactose (Gal), glucose (Glu), inositol (Ino), sucrose (Suc), maltose (Mal), and trehalose (Tre). The results showed distinct differences in Ara, Rha, Fru, Fuc, Gal, Glu, Ino, and Tre between the high and common resin yields. Compared with the common resin yield, Ara, Fuc, Gal, Glu, and Ino accumulated more, whereas Rha, Fru, and Tre dramatically decreased in the high one. Sucrose content demonstrated an increasing tendency in the high resin yield, despite no statistically notable difference being investigated between them.

### 2.3. Differences in Endogenous Hormone Levels between High and Common Resin Yield

**Figure 3:**
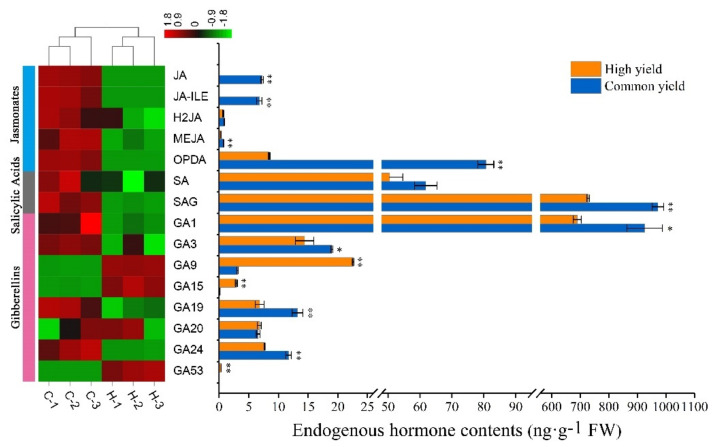
Endogenous hormone levels of masson pine needles in the high and common resin yield. FW means fresh weight. “Common yield” means the common resin-yielding clone, and “High yield” means the high resin-yielding clone. Error bars refer to the standard deviation (SD) of three biological repeats. * and ** indicate significant differences between the high and common ones at *p* < 0.05 and *p* < 0.01, respectively. C-1, C-2, and C-3 represent three biological repeats of the common one, H-1, H-2, and H-3 represent three biological repeats of the high one. The color brightness represents the degree of the content difference, as shown in the color bar. GA15 is too small to be seen and GA53 is too low to be detected in the common one, and JA, as well as JA-ILE, are too low to be detected in the high one. JA, jasmonic acid; JA-ILE, jasmonoyl-L-isoleucine; H2JA, dihydrojasmonic acid; MEJA, methyl jasmonate; OPDA, cis (+)-12-oxophytodienoic acid; SA, salicylic acid; SAG, salicylic acid 2-O-β-glucoside; GA1, gibberellin A1; GA2, gibberellin A2; GA3, gibberellin A3; GA4, gibberellin A4; GA7, gibberellin A7; GA9, gibberellin A9; GA15, gibberellin A15; GA19, gibberellin A19; GA20, gibberellin A20; GA24, gibberellin A24; GA53, gibberellin A53.

The hormones JAs, SAs, and GAs involved in plant secondary metabolism were quantified so as to unravel the accumulation characteristics of endogenous hormones between the high and common resin yield, and the significant differences in these hormones were investigated. GA9, GA15, and GA53 were highly increased, while JA, JA-ILE, MeJA, OPDA, SAG, GA1, GA3, GA19, and GA24 were dramatically decreased in the high resin yield in comparison with those in the common one. SA level showed a decreasing tendency in the high resin yield, despite no statistically obvious difference being investigated between them (Figure 3).

### 2.4. Full-Length Transcriptome Profiling and DEGs Identification

The full-length transcriptome data were obtained based on the third-generation sequencing platform of PacBio Sequel. By filtering the raw data, removing the connector and original offline data that was less than 50 bp in length, 15,216,993 subreads with a data volume of 23.92 G were obtained (Table 1). The average length of the Subreads was 1572 bp. A total of 520,718 circular consensuses (CCS) were obtained through conditional screening (full passes of 1). Subsequently, 416,262 full-length non-chimeric reads (FLNC) were obtained with complete 5′-primers, 3′-primers, and poly-A tails in which an average of the FLNC read length was 1719. The hierarchical n*log(n) algorithm was used to cluster the FLNC sequences to obtain cluster consensus. Finally, arrow software was used to correct cluster consensus, generating 38,784 high-quality polish consensus transcripts for further analysis. The third-generation sequencing technology represented by PacBio has the advantage of ultralong read length, while the technique has a high single base error rate. To reduce the high error rate and improve the sequencing accuracy, the Illumina short reads were used for correction. After correcting the data, 38,784 consensuses, 2142 N50, and 1138 N90 were obtained. The corrected transcripts were aligned and clustered with CD-HIT software, and then removed redundant and similar sequences that resulted in 38,784 non-redundant transcripts and 17,266 genes.

To obtain a comprehensive gene functional annotation, a total of 17,266 genes were analyzed using Nr, Nt, Pfam, KOG, Swiss-Prot, KEGG, and GO. The results showed that 8200 genes were simultaneously annotated in all the databases (Nr, Nt, Pfam, GO, KEGG, KOG, and Swiss-Prot) (Figure S1A). There were 16,973 genes annotated in at least one database of which 16,678 (96.59%) genes were annotated by the Nr database. The alignment of sequence homology demonstrated that 8913 (51.62%) sequences were found against *Picea sitchensis*, 1897 (10.99%) sequences had significant hits for *Amborella trichopoda*, then followed by *Nelumbo nucifera* (1105, 6.40%), *Marchantia polymorpha* (351, 2.03%), *Elaeis guineensis* (282, 1.63%), and 23.57% of the sequences were homologous to other species (Figure S1B). A total of 12,187 (70.58%) genes were annotated in the GO database. The results of the GO enrichment analysis showed that genes were mainly enriched in metabolic process, cellular process, single-organism process, cell, cell part, organelle, binding, catalytic activity, transporter activity in biological process (BP), molecular function (MF), and cellular component (CC) (Figure S1C). A total of 16,284 genes were annotated in the KEGG database. The top three KEGG pathways were “Metabolism, Carbohydrate Metabolism” (771, 4.47%), “Environmental Information Processing, Signal Transduction” (858, 4.97%), and “Genetic Information Processing, Translation” (740, 4.29%) (Figure S1D).

**Figure 4:**
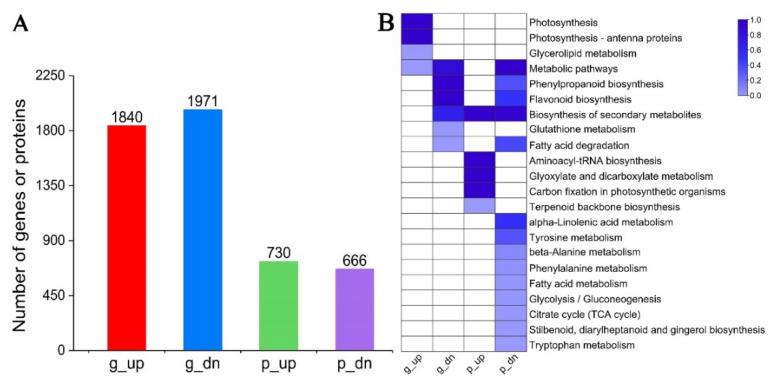
Identification of DEGs/DEPs. (**A**) Number of DEGs/DEPs. (**B**) Functional category enrichment of DEGs/DEPs. DEGs/DEPs were classified into two groups of up-regulated or down-regulated, without DEGs and DEPs comparison. The degree of the pathway enrichment was indicated according to the value of −log(*q*-value), as shown in the color bar. Each group was indicated by expression level (“g” for gene and “p” for protein, respectively) and regulatory direction (“up” for up-regulated and “dn” for down-regulated, respectively). The color brightness represents the degree of significant enrichment of pathways, as shown in the color bar.

In total, 3811 DEGs were identified in needles. To reveal the metabolic pathways related to the resin yield, these DEGs were divided into two groups of up- and down-regulated, and then functional category enrichment analysis was performed for each group, respectively. There were 1840 up-regulated and 1971 down-regulated DEGs (Figure 4A). These up-regulated DEGs were significantly enriched in photosynthesis, photosynthesis-antenna proteins, glycerolipid metabolism, and metabolic pathways. In contrast, those down-regulated DEGs were significantly enriched in phenylpropanoid biosynthesis, flavonoid biosynthesis, metabolic pathways, biosynthesis of secondary metabolites, glutathione metabolism, and fatty acid degradation (Figure 4B).

To validate the RNA-Seq results, a total of 12 genes, which were involved in terpenoids biosynthesis, JA biosynthesis & signaling, photosynthesis, and carbohydrate metabolism, were tested by the qRT-PCR. The results of qRT-PCR showed that expression levels of all selected genes were consistent with RNA-Seq data, indicating that transcriptome data are reliable (Figure S2).

### 2.5. Proteome Profiling and DEPs Identification

In this study, 68,801 spectra were matched by mass spectrometry. In total, 27,934 peptides, 22,031 unique peptides and 4855 proteins were identified with a 5% FDR. Of the 4855 proteins identified, a total of 4566 proteins were confidently identified and quantified (Figure S3A). The distribution of unique peptides defining each protein is shown in Figure S3B, with over 74% of them, including at least two peptides. Peptide coverage is shown in Figure S3C, with 0–5% and 20–30% coverage having the highest number of proteins. The molecular mass of the identified proteins is shown in Figure S3D, more than 60% of the proteins had masses between 20 and 60 kDa.

In total, 1396 DEPs were identified, of which 730 were up-regulated and 666 were down-regulated (Figure 4A). Functional enrichment analysis demonstrated that these up-regulated proteins were significantly enriched in aminoacyl-tRNA biosynthesis, glyoxylate dicarboxylate metabolism, carbon fixation in photosynthetic organisms, biosynthesis of secondary metabolites, and terpenoid backbone biosynthesis, whereas these down-regulated proteins were significantly enriched in metabolic pathways, biosynthesis of secondary metabolites, alpha-Linolenic acid metabolism, flavonoid biosynthesis, fatty acid degradation, phenylpropanoid biosynthesis, tyrosine metabolism, beta-Alanine metabolism, phenylalanine metabolism, fatty acid metabolism, glycolysis/gluconeogenesis, citrate cycle, stilbenoid, diarylheptanoid and gingerol biosynthesis, and tryptophan metabolism (Figure 4B).

### 2.6. Correlation of mRNA and Protein Profiles

**Figure 5:**
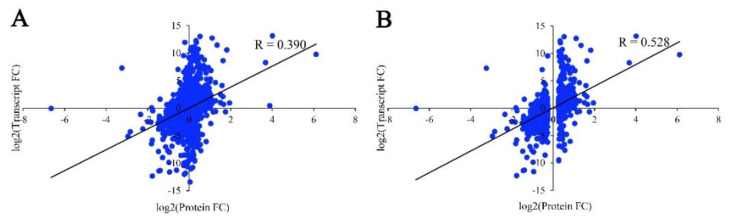
Correlation between the expression of proteins and mRNAs. (**A**) Correlation of all quantified proteins and their corresponding mRNAs. (**B**) Correlation of DEPs and their corresponding mRNAs. “R” represents the correlation coefficient calculated by the Pearson correlation test, and “FC” represents fold change.

A correlation analysis was conducted between the transcriptomic and proteomic data. Correlation analysis indicated a low correlation coefficient (R = 0.390, *p* < 0.001) between the expression levels of all quantified proteins and their corresponding mRNAs. However, an about 1.4-fold correlation was revealed between the DEPs and their corresponding mRNAs (R = 0.528, *p* < 0.001) (Figure 5), suggesting a certain degree of the biological relevance of protein and mRNA changes in relation to the resin yield.

Overall, 3811 DEGs, and 1396 DEPs, were identified in needles. Among these DEGs and DEPs, 411 were commonly regulated both at the mRNA and protein levels. Of these, 361 were regulated with the same trend, suggesting that the expression changes of these proteins are mainly controlled by transcriptional alterations. However, there were a few genes, that were changed with the opposite trend at the transcriptional and proteomic levels, including acetyl-CoA C-acetyltransferase (*AACT*) involved in terpenoid backbone biosynthesis, linoleate 9S-lipoxygenase (*LOX1*) related to linoleic acid metabolism, ribose 5-phosphate isomerase A (*RPIA*) and phosphoserine phosphatase (*PSPH*) associated with biosynthesis of amino acids, and 25.3 kDa heat shock protein (*HSP25.3*) referred to protein processing in the endoplasmic reticulum (Table S2), suggesting the involvement of post-transcriptional regulation for these genes/proteins. Therefore, the integrative analysis of proteome and transcriptome would provide more information on the genes involved in the resin yield in masson pine.

### 2.7. Integrated Analysis of Transcriptome and Proteome

**Figure 6:**
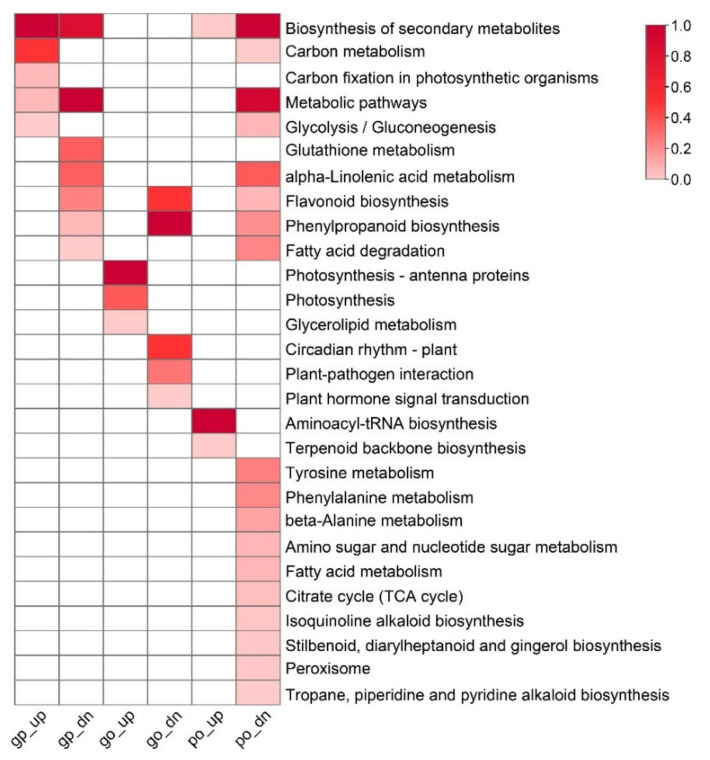
Functional category enrichment of DEGs/DEPs. DEGs/DEPs were classified into a total of 6 groups according to RNA-Seq and iTRAQ integrative analysis. The degree of the pathway enrichment was indicated according to the value of −log(*q*-value), as shown in the color bar. Each group was indicated by expression level (“go” for gene only, “po” for protein only, and “gp” for both gene and protein level, respectively) and regulatory direction (“up” for up-regulated and “dn” for down-regulated, respectively). The color brightness represents the degree of significant enrichment of pathways, as shown in the color bar.

By comparing the DEGs with the DEPs, there were 156 up-regulated and 205 down-regulated genes changed with the same trend both at the mRNA and protein levels (Figure S4). These up-regulated genes were significantly enriched in the biosynthesis of secondary metabolites, carbon metabolism, carbon fixation in photosynthetic organism/Calvin cycle, metabolic pathways, and glycolysis/gluconeogenesis (Figure 6). It is noteworthy that one monoterpene synthase-like alpha-terpineol synthase (*MonoTPS1*), together with two sesquiterpene synthase-like caryophyllene/humulene synthase (*SesquiTPS1*) and 5-germacradien-4-ol synthase (*SesquiTPS2*) were dramatically up-regulated, indicating their crucial roles in the resin yield in masson pine. Consistently, photosystem II protein *PsbR* and ribulose-1,5-bisphosphate carboxylase/oxygenase small subunit (*rbcS*), together with F-type H+-transporting ATPase subunit delta (*ATPD*) involved in photosynthesis and chlorophyll synthase (*chlG*) involved in chlorophyll biosynthesis were significantly up-regulated. Besides, five genes, hexokinase (*HXK*), sucrose synthase (*SUS*), phosphoglycerate kinase (*PGK*), dihydrolipoamide dehydrogenase (*PDH*), and dihydrolipoamide succinyltransferase (*DLST*) involved in carbohydrate metabolism, were also obviously up-regulated (Table S3). In contrast, these down-regulated genes were significantly enriched in metabolic pathways, biosynthesis of secondary metabolites, glutathione metabolism, alpha-Linolenic acid metabolism/JA biosynthesis, flavonoid biosynthesis, phenylpropanoid biosynthesis, and fatty acid degradation (Figure 6). Notably, sesquiterpenoid biosynthesis-related genes like (E)-alpha-bisabolene synthase (*SesquiTPS3*) and MVA pathway-related genes like phosphomevalonate kinase (*PMK*) and diphosphomevalonate decarboxylase (*MVD*) were significantly down-regulated. Consistently, flavanone 3-hydroxylase (*F3H*), bifunctional dihydroflavonol 4-reductase (*DFR*), anthocyanidin synthase (*ANS*), and anthocyanidin reductase (*ANR*) participated in flavonoid biosynthesis, together with caffeoyl-CoA O-methyltransferase (*CCoAOMT*) and caffeic acid 3-O-methyltransferase (*COMT*) participated in phenylpropanoid biosynthesis were also obviously down-regulated. Totally three JA biosynthesis genes, of which 12-oxophytodienoic acid reductase (*OPR*) and two jasmonate O-methyltransferases (*JMTs*), were significantly down-regulated. In addition, glutathione reductase (*GSR*) and glutathione S-transferases (*GST*) referred to as glutathione metabolism were also notably down-regulated (Table S3).

Since only a few genes were significantly altered both at the transcriptomic and proteomic levels, similar functional enrichment results were obtained for the up-and down-regulated genes with or without removing those genes that were significantly altered at the proteomic level and also for the up- and down-regulated proteins with or without removing those proteins that were significantly altered at the transcriptomic level (Figure 4B and Figure 6). Next, we primarily focused on the genes/proteins regulated only at the transcriptomic or proteomic level in specific pathways.

There were 1666 up-regulated and 1734 down-regulated DEGs whose corresponding proteins were not differentially expressed (Figure S4). Consistent with the results of functional enrichment, the expression levels of genes involved in photosynthesis were significantly elevated, including photosystem I subunit *PsaE*, *PsaF*, *PsaG*, *PsaH*, *PsaL*, and *PsaO*, photosystem II protein *PsbP*, *PsbS*, and *PsbY*, chlorophyll a/b binding protein *Lhca1*, *Lhca3*, *Lhcb1*, *Lhcb2*, and *Lhcb4*, and Calvin cycle-related genes like *rbcS*, glyceraldehyde 3-phosphate dehydrogenase (*GAPDH*) and fructose-1,6-bisphosphatase I (*FBP1*). Accordingly, the expression levels of genes related to chlorophyll biosynthesis, including porphobilinogen synthase (*HemB*), protoporphyrinogen/coproporphyrinogen III oxidase (*HemY*), and magnesium-protoporphyrin O-methyltransferase (*chlM*), were notably elevated. Consistently, the expression levels of genes associated with terpenoid biosynthesis were also greatly elevated, including MVA pathway-related genes like farnesyl diphosphate synthase (*FPPS*), MEP pathway-related genes like geranyl diphosphate synthases (*GPPS1* and *GPPS2*), and monoterpene synthase-like beta-pinene synthase (*MonoTPS2*). In addition, sucrose-phosphate synthase (*SPS*) referred to as sucrose biosynthesis and 6-phosphofructokinase (*PFK*) referred to as glycolysis were also dramatically up-regulated. Interestingly, several genes related to circadian rhythm-plant, including phytochrome-interacting factor 3 (*PIF3*), casein kinase II subunit alpha (*CSNK2A*), cryptochrome 1 (*CRY1*), and transcription factor HY5 (*HY5*), were obviously up-regulated (Table S3). On the contrary, the expression levels of genes involved in phenylpropanoid biosynthesis significantly declined, including phenylalanine ammonia-lyase (*PAL*), 4-coumarate-CoA ligase (*4CL*), and caffeic acid 3-O-methyltransferas (*COMT*). Consistently, the expression levels of genes that participated in flavonoid biosynthesis, including chalcone synthase (*CHS*), flavonol synthase (*FLS*), *DFR*, *ANS*, and *ANR*, were greatly downregulated. Besides, GA biosynthesis genes including ent-copalyl diphosphate synthase 1 (*CPS1*) and ent-kaurene oxidase 1 (*KO1*) as well as JA signaling genes including three coronatine-insensitive proteins 1 (*COI1s*) and two jasmonate ZIM domain-containing protein (*JAZs*) were significantly down-regulated (Table S3).

There were 542 up-regulated and 443 down-regulated DEPs whose corresponding genes were not differentially expressed (Figure S4). As expected, proteins involved in the MEP pathway were significantly up-regulated, including 1-deoxy-D-xylulose 5-phosphate reductoisomerase (DXR), 4-diphosphocytidyl-2-C-methyl-D-erythritol kinase (CMK), 2-C-methyl-D-erythritol 2,4-cyclodiphosphate synthase (MDS), 4-hydroxy-3-methylbut-2-en-1-yl diphosphate reductase (HDR1), and geranylgeranyl diphosphate synthase (GGPPS1). Accordingly, proteins related to terpenoid biosynthesis were greatly up-regulated, including monoterpenoid biosynthesis-related proteins like alpha-pinene synthase (MonoTPS3), diterpenoid biosynthesis-related proteins like monofunctional isopimaradiene synthase (DiTPS), and diterpene resin acid biosynthesis-related proteins like cytochrome P450 720Bs (CYP720B8, CYP720B10, and CYP720B11). Consistent with the MEP pathway, proteins associated with photosynthesis were dramatically up-regulated as well, including photosystem I subunit PsaK, and photosystem II protein Psb28, rbcS, ribulose-1,5-bisphosphate carboxylase/oxygenase large subunit (rbcL) and glyceraldehyde 3-phosphate dehydrogenase A subunit (GAPA). Similarly, many proteins that participated in aminoacyl-tRNA biosynthesis were also notably up-regulated. Several proteins referred to as chlorophyll biosynthesis, including glutamate-1-semialdehyde 2,1-aminomutase (HemL), hydroxymethylbilane synthase (HemC), magnesium chelatase subunit (chlH), magnesium-protoporphyrin IX monomethyl ester (oxidative) cyclase (chlE), and protochlorophyllide reductase (POR), were obviously up-regulated. In addition, proteins involved in protein processing in the endoplasmic reticulum were significantly up-regulated. Most of these proteins belonged to HSPs, including HSP20, HSP90B, and HSPA1s (Table S3).

**Figure 7:**
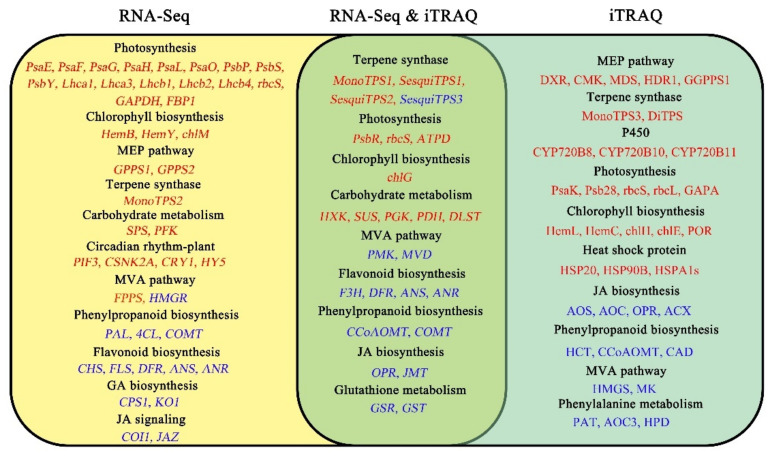
Representative pathways and candidate genes are significantly associated with the resin yield by RNA-Seq and iTRAQ integrative analysis. Up- and down-regulated DEGs/DEPs were indicated by red and blue colors, respectively.

In contrast, the expression levels of proteins involved in JA biosynthesis were greatly decreased, including hydroperoxide dehydratase (AOS), allene oxide cyclase (AOC), 12-oxophytodienoic acid reductase (OPR), and acyl-CoA oxidase (ACX). Accordingly, two proteins referred to the MVA pathway, including 3-hydroxy-3-methylglutaryl-CoA-synthase (HMGS) and mevalonate kinase (MK), were significantly down-regulated. Similarly, proteins that participated in phenylpropanoid biosynthesis including shikimate O-hydroxycinnamoyltransferase (HCT), CCoAOMT, and cinnamyl-alcohol dehydrogenase (CAD), were also obviously decreased. Besides, several proteins associated with phenylalanine metabolism were notably down-regulated, including aspartate aminotransferase (PAT), primary-amine oxidase (AOC3), and 4-hydroxyphenylpyruvate dioxygenase (HPD) (Table S3). Ultimately, these pathways significantly changed in high and common resin yield through RNA-Seq and iTRAQ integrative analysis were summarized in Figure 7.

### 2.8. Identification of Key Genes at the Transcriptomic, Proteomic and Biochemical Levels

Some important genes with the same trend at the transcriptomic and proteomic levels were revealed by transcriptomic and proteomic integrative analysis, which mainly involved in the biosynthesis of secondary metabolites, carbon metabolism, Calvin cycle, glycolysis, JA biosynthesis, glutathione metabolism, phenylpropanoid biosynthesis, flavonoid biosynthesis, and fatty acid degradation (Figure 6). At the biochemical level, there were notable differences in the contents of photosynthetic pigment, sugar, and hormone between the high and common resin yields. These biochemical variations were consistent with the changes in the expression levels of genes involved in chlorophyll biosynthesis, carbohydrate metabolism, and JA biosynthesis, strongly indicating the involvement of these genes in resin yield. Thus, the genes with the same trend at the transcriptomic, proteomic and biochemical levels could be used as markers for the high resin yield, including *ChlG*, *HXK*, *SUS*, *PGK*, *PDH*, *DLST*, *OPR*, and *JMT*. According to the expression levels of these genes, *ChlG*, *HXK*, *SUS*, *PGK*, *PDH*, and *DLST* were a positive correlation with resin yield, whereas *OPR* and *JMT* were a negative correlation with resin yield.

## 3. Discussion

### 3.1. Identification of DEGs and DEPs

RNA-Seq and iTRAQ methods have extensively been used to identify DEGs and DEPs involved in plant development and secondary metabolism. In recent years, many RNA-Seq studies have been widely conducted to investigate the transcriptional regulation in the resin yield of masson pine [36,37,38,39,40]. However, the application of iTRAQ-based proteomic analysis was relatively rare, so far, no report was available to explore the molecular regulatory mechanisms in resin yield of masson pine using these two approaches simultaneously. To better understand the molecular regulatory mechanisms of resin yield and to identify the key genes and pathways at the transcriptomic and proteomic levels, in this study, Iso-Seq, RNA-Seq, and iTRAQ were performed in parallel to investigate the DEGs and DEPs from needles of masson pine with the high and common resin yield. In total, 17,266 genes and 4566 proteins were quantified. However, a poor correlation (R < 0.40) was revealed between these quantified proteins and their corresponding mRNAs, consistent with the results of previous studies comparing protein and mRNA expression levels [41,42,43]. Notably, the correlation coefficient was higher between the expression levels of DEGs and DEPs (Figure 5), suggesting the involvement of consistency changes of DEGs and DEPs in resin yield in masson pine. Comparative analysis of DEGs and DEP revealed that a few genes were commonly regulated both at the transcriptomic and proteomic levels, indicating that the proteins showed significant expression changes and did not always have a corresponding change at the transcriptional level. The poor overlap between DEGs and DEPs also demonstrated that post-transcriptional regulation plays an important role in the resin yield of masson pine. Among these overlapped DEGs and DEPs, the functional categories were related to secondary metabolism biosynthesis, then followed by carbon metabolism, Calvin cycle, glycolysis, JA biosynthesis, and glutathione metabolism, indicating their involvement in the resin yield of masson pine.

Many genes involved in carbohydrate metabolism among these overlapping DEGs and DEPs exhibited the same trend at the transcriptomic, proteomic and biochemical levels, indicating their contribution to the resin yield of masson pine. Previous studies on rubber trees showed that carbohydrate metabolism has a close relationship with rubber yield [44]. In masson pine, studies revealed that the resin-producing capacity reached highly significant levels in relation to soluble sugar content [45]. The results of sugar detection showed that sucrose was the most abundant sugar in the needles of masson pine, then followed by glucose and fructose, and these three soluble sugars accounted for 90% of all sugars. Compared with the common resin yield, the accumulation of more sucrose and glucose could provide more raw materials for resin biosynthesis in the high resin yield. Several key genes involved in carbohydrate metabolism, including *SUS*, referred to sucrose synthesis, *HXK* and *PGK* referred to glycolysis, and *PDH* as well as *DLST* referred to the TCA cycle, were significantly up-regulated at the transcriptomic and proteomic levels, indicating that the improvement of the expression levels of these key genes was beneficial to enhance the resin biosynthesis and increase the resin yield in masson pine.

### 3.2. Roles of Terpenoid Biosynthesis Genes in Resin Yield

**Figure 8:**
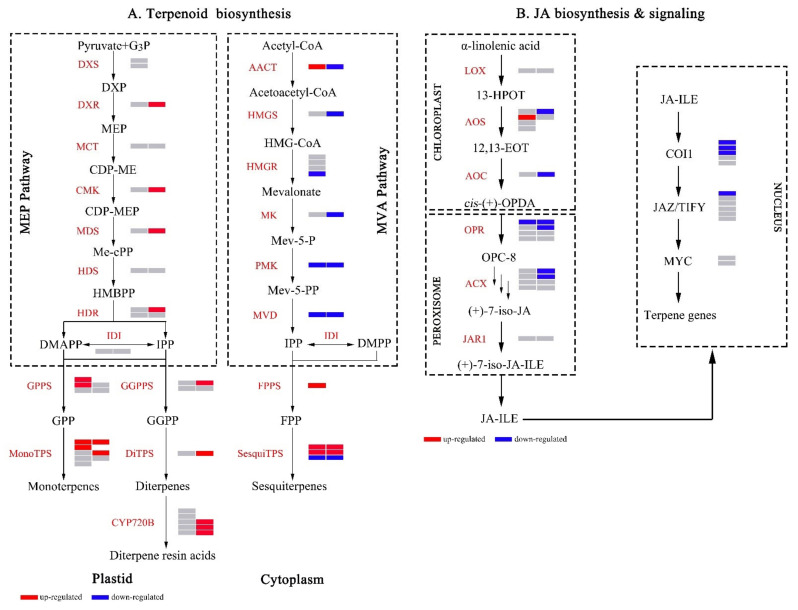
Heatmap of DEGs and DEPs related to terpenoid biosynthesis (**A**), JA biosynthesis & signaling (**B**). Up- and down-regulated DEGs/DEPs were indicated by red and blue colors, respectively. Nonsignificant genes/proteins were indicated by a gray color. The cells (from left to right) in the heatmap represent the expression change of gene and protein, respectively. The deletion of the cells on the right represents that the protein corresponding to the gene was not detected in the iTRAQ experiment.

Resin is composed of terpenoids, and terpenoid biosynthesis plays a central role in the resin yield. The resin biosynthesis of conifer derives from the two isoprenoid molecules isopentenyl diphosphate (IPP) and dimethylallyl diphosphate (DMAPP) which originate from the MEP and MVA pathways. Two or more of these five-carbon (C5) metabolites are conjugated by isoprenyl diphosphate synthases (IDS) to produce the longer-chain (C10, C15, C20) substrates for TPS. MEP and MVA pathway genes and their functions are extensively conserved [46], and many genes of these pathways have been described in conifers. In this study, a total of 25 genes, that covered the entire MEP and MVA pathways were identified (Figure 8A). The majority of genes involved in MEP and MVA pathways exhibited significant changes at the transcriptional and proteomic levels. In the MEP pathway, the majority of genes were significantly up-regulated at the proteomic level (including *DXR*, *CMK*, *MDS*, *HDR1*, and *GGPPS1*), and two *GPPSs* (including *GPPS1* and *GPPS2*) and *FPPS* were greatly up-regulated at the transcriptional level, indicating that increased expression levels of genes involved in MEP pathway contributed to the increase of resin yield. However, most members in the MVA pathway exhibited different changes from the MEP pathway. The majority of genes related to the MVA pathway were dramatically down-regulated at the transcriptional and proteomic levels (including *HMGS*, *HMGR4*, *MK*, *PMK*, and *MVD*). The above results indicated that the MEP pathway played a more important role in the increase of resin yield than the MVA pathway. Remarkably, *PMK* and *MVD* showed consistent expression changes both at the mRNA and protein levels, indicating that these two genes were a negative correlation with resin yield. *IDSs* (including *GPPS*, *FPPS*, and *GGPPS*), which produce GPP, FPP, and GGPP as precursors of monoterpenoids, sesquiterpenoids, and diterpenoids, respectively, are key genes for terpenoid biosynthesis. These genes were greatly up-regulated in high resin yield, which was consistent with previous reports [36,37], indicating their involvement in resin yield.

The TPS generates much of the huge chemical diversity found in conifer resin [5,47,48]. Conifer TPS genes include *MonoTPS*, *SesquiTPS*, and *DiTPS*, which use DMAPP, GPP, FPP, and GGPP, respectively, as substrates for resin. In the present study, we found five *MonoTPSs*, three *SesquiTPSs*, and one *DiTPS* in the needles, and *MonoTPSs* and *SesquiTPSs* exhibited greater differences than *DiTPS* between high and common resin yield, indicating that there were significant differences in the monoterpene and sesquiterpene biosynthesis in needles from high and common resin yield. Cytochrome P450 enzymes of the CYP720B subfamily play a central role in the DRAs biosynthesis, which has been identified in different spruce and pine genomes and transcriptomes. The known CYP720B sequences are classified into four distinct clades I–IV. Of these known spruce and pine CYP720Bs, four have been proven to be involved in the biosynthesis of DRAs, two in clade I (CYP720B2, CYP720B12) and two in clade III (CYP720B1, CYP720B4) [49,50,51]. In a recent study, the *PmCYP720B11v2* gene was also proven to be involved in the metabolic processes of diterpene biosynthesis in the stem as a positive part of the defense against pinewood nematode (PWN) attack in masson pine [52]. In the present study, three genes in clade I (*CYP720B2*, *CYP720B10*, *CYP720B11*), one gene in clade III (*CYP720B1*), and one gene in clade IV (*CYP720B8*) were identified in needles. These *CYP720B* genes did not exhibit significant differences at the transcript level, only *CYP720B8*, *CYP720B10*, and *CYP720B11* differed at the protein level, indicating that there were no significant differences in DRAs biosynthesis in needles from high and common resin yield. The previous works revealed that *CYP720Bs* exhibited higher expression in roots and stems, and the contents of DRAs were higher in roots and stems than those in needles [53,54], which would be the explanation for the lack of significant differences in DRAs biosynthesis in needles from high and common resin yield.

### 3.3. Roles of Photosynthesis Genes in Resin Yield

As one of the by-products of photosynthesis, the resin is a way of carbon fixation in pine trees, so photosynthesis is a crucial physiological process that determines the resin yield [15]. Photosynthesis affects the resin yield in two main aspects, one is whether the photosynthetic products are sufficient for resin biosynthesis, and the other is whether the pine trees can grow vigorously to develop enough resin ducts [55]. Previous studies revealed that the photosynthetic capacity of the high resin-yielding clone is much stronger than that of the low resin-yielding clone, and self-defense measurement can be quickly initiated under strong light and high temperature in high resin yield [16]. In this study, many genes involved in photosynthesis were significantly up-regulated at the transcriptional and proteomic levels. LHCs in PSI and PSII play crucial roles in light harvesting and light protection [56,57]. Several genes referred to LHCs, such as *Lcha1*, *Lcha3*, *Lchb1*, *Lchb2*, and *Lchb4*, were greatly up-regulated at the transcriptional level, indicating that high resin yield had a stronger capacity for light-harvesting and light protection, which could dissipate excess excitation energy under strong light and maintain efficient photosynthetic capacity under strong light. PSII is a protein complex composed of multiple subunits, and PsbR is a small-molecule subunit protein of PSII, which plays an important role in assisting the stable assembly of the PSII core complex and water oxidation [58]. In this study, the *PsbR* gene was greatly up-regulated both at the mRNA and protein levels. In addition, the PsbS may stabilize the PSII–LHCII supercomplex structure and improve electron transmission efficiency [59], our results showed that the *PsbS* gene was significantly up-regulated at the proteomic level. ATPD is an F-type ATP synthase present in the chloroplast, which utilizes the transmembrane proton electrochemical gradient formed in electron transport to catalyze the synthesis of ATP from ADP and convert light energy into chemical energy [60]. In the present study, the *ATPD* gene was notably up-regulated both at the mRNA and protein levels, suggesting that high resin yield had higher ATPase activity and could generate more ATP for the biosynthesis of secondary metabolites. Rubisco (rbcL and rbcS) is a bifunctional enzyme located in the chloroplast stoma and catalyzes photosynthetic CO_2_ fixation to form ribulose-1,5-bisphosphate (RuBP) [61], and the transcript levels of *rbcL* and *rbcS* could directly reflect the photosynthetic efficiency [62]. In this study, *rbcS* was up-regulated both at the transcriptional and proteomic levels, and *rbcL* was up-regulated at the proteomic level, indicating that the up-regulation of the *rbcL* and *rbcS* might play a crucial role in resin yield. These results suggested that up-regulation of the Rubisco-related genes in high resin yield enhanced CO_2_ assimilation, which played an important role in the production of photosynthetic products from needles and provides a material basis for growth and the biosynthesis of secondary metabolites in masson pine.

Chlorophyll is an essential pigment for photosynthesis in green plants and is crucial for light absorption, energy transfer, and photosynthetic charge separation that eventually result in the transfer of an electron from the photosystem to NADP+ and proton-driven ATP production [63]. Previous works revealed a significant correlation between chlorophyll contents and resin yield in masson pine, and the chlorophyll contents of high resin yield were significantly higher than those of common resin yield [64]. Consistently, in our study, the chlorophyll contents were dramatically increased in high resin yield in comparison with that in the common one. High chlorophyll contents would be beneficial for harvesting light energy and its conversion to chemical energy [65]. The variations in chlorophyll contents between high and common resin yield demonstrated that there might be great differences in photosynthetic capacity between them. Chl b is a prerequisite for the stable existence of light-harvesting complex protein (LHCP) and the increase of Chl b was found to be beneficial for the absorption of short-wavelength light, thus effectively improving the light energy utilization efficiency of plants under low light [66]. Previous studies suggested that Chl b could switch from an active photochemical state to a photoprotective state [67]. In the present study, the Chl b content was greatly increased in high resin yield in comparison with that in the common one, which was beneficial to the stability of needle LHCP, thus enhancing the utilization of light energy under low light. Moreover, the higher Chl b content of high resin yield indicated that the photosynthetic protection mechanism was more responsive than that of common resin yield. This could also be supported by the Car content, which is involved in several photoprotection mechanisms [68], sharing the same pattern of variations. Further, we focused on the expression of chlorophyll synthesis genes in high resin yield. Consistent with the changes in chlorophyll contents, many key genes involved in chlorophyll biosynthesis were significantly up-regulated at the transcriptional or proteomic level, including *HemL*, *HemB*, *HemC*, *HemY*, *chlH*, *chlM*, *chlE*, *POR*, and *chlG*. Of which, the chlG enzyme catalyzes chlorophyllide a (chlide a) and chlorophyllide b (chlide b) to Chl a and Chl b, the reaction is the last step in chlorophyll biosynthesis, the enzyme is essential for the formation of chromoprotein complexes and stabilization of chlorophyll-binding proteins [69]. Furthermore, *chlG* is involved in the feedback regulation of chlorophyll biosynthesis and plays an important role in the stable assembly of chlorophyll-binding proteins and other thylakoid membrane components [63,70]. In the present study, *HemB*, *HemY*, and *ChlM* were up-regulated at the transcriptional level; *HemL*, *HemC*, *chlH*, *chlE*, and *POR* were up-regulated at the proteomic level. It is worth noting that *chlG* was significantly up-regulated both at the transcriptional and proteomic levels. Therefore, the improved expression of multiple genes related to chlorophyll biosynthesis facilitated the increase of chlorophyll contents, leading to the enhancement in photosynthetic efficiency, thus, contributing to the accumulation of photosynthetic products and the increase of resin yield.

### 3.4. Roles of Hormone Biosynthesis Genes in Resin Yield

Plant hormones are key players involved in the biosynthesis of secondary metabolites [71,72]. Jasmonates (JAs), as an important endogenous signaling molecule of hydroxyl lipids, are widely involved in plant growth and development, metabolic regulation, stress response, and defense response [73,74]. When plants are injured, JAs will respond quickly and induce the expression of defense genes and the biosynthesis of secondary metabolites to obtain defense against pests and diseases [75]. In the current study, we observed that JAs (including JA, JA-ILE, MEJA, and OPDA) levels were greatly decreased in high resin yield comparison with those in the common one. (Figure 3). Consistent with the changes in JA levels, many key genes that participated in JA biosynthesis were significantly down-regulated at the protein level (including *AOS*, *AOC*, *OPR*, *ACX*, and *JMT*) (Figure 8B), strongly indicating their involvement in the regulation of resin yield. We supposed that there might be a negative feedback regulation of JA-dependent resin biosynthesis in the formation of resin yield. The resin biosynthesis capacity was stronger in the high resin yield than that in the common one, so the lower JAs levels could maintain the normal resin biosynthesis, which might be the explanation for the lower JAs levels in high resin yield. *COI1* and *JAZ* play a crucial role in JA signaling, notably, *JAZ* acts as a ‘suppressor’ in JA signaling pathway [76,77]. In this study, *COI1* and *JAZ* were greatly down-regulated at the transcript level, indicating that *COI1* and *JAZ* might negatively regulate rein yield in masson pine.

In addition to JAs, it is noteworthy that other hormones such as SAs and GAs are also involved in secondary metabolism [78,79]. In this study, SA level demonstrated a decreased tendency in the high resin yield, despite no statistically significant difference being investigated between them. Several genes involved in phenylalanine metabolism, including *PAT*, *AOC3*, and *HPD*, were significantly decreased at the protein level, in accord with the decrease of SA and SAG levels. These results indicated the possible involvement of SA in the regulation of resin yield. The GA and terpenoid biosynthesis both use GGPP as a common precursor [80]. In the present study, GAs levels were dramatically different between high and common resin yields. It is worth noting that GA1 content was the highest among all GAs measured, and its content greatly declined in high resin yield. Consistent with the decrease of GA levels, the transcripts of several key genes referred to as GA biosynthesis (including *CPS1* and *KO1*) were obviously down-regulated. Our results demonstrated that high resin yield could provide more substrates (GGPP) for resin terpenoid biosynthesis by down-regulating GA biosynthesis. Together, these findings revealed that JA, SA, and GA might play an important role in the regulation of resin yield in masson pine.

## 4. Materials and Methods

### 4.1. Plant Materials and Growth Conditions

The experiments were performed using 12-year-old masson pine clones cultivated in seed orchards established in 2007 by Nanning Forestry Research Institute (23°10′ N, 108°00′ E), Wuming County, Guangxi, China. The sample collection site belongs to the subtropical monsoon climate, with sufficient light and heat, and abundant rainfall. The annual average temperature is 21.7 °C, the highest temperature is 40.7 °C, the lowest temperature is as low as −0.8 °C, and the annual average rainfall is 1100–1700 mm. The resin yield was measured by the bark streak method of wounding for resin tapping for three consecutive years [14] and calculated as the yield of the individual per day per 10 cm width of cutting surface in grams to adjust the effect of stem diameter on resin yield. Based on the measurement, two groups of trees were selected, including a high resin-yielding clone (15.96 g·d^−1^·10 cm^−1^) and a common resin-yielding clone (8.18 g·d^−1^·10 cm^−1^). Current year needles from the high resin-yielding clone and common resin-yielding clone in August (autumn) were harvested for biochemical, transcriptomic, and proteomic analyses. The common resin-yielding clone served as the control. Needles obtained from four orientations at the same height with equal volume for each tree were mixed into one sample, and three trees for each clone were selected as three biological replicates.

### 4.2. Detection of Photosynthetic Pigment Contents

The fresh needles from six independent samples were collected using self-sealing bags and refrigerated until the analysis. The photosynthetic pigment contents were measured using the acetone extraction method [81]. The sample was weighed to 0.1 g (accurate to 0.001 g), and pure acetone was added to reach a volume of 10 mL. The supernatant was extracted until the needles completely turned white in darkness at room temperature. The absorbance values at 470, 645, and 662 nm were determined to assess the photosynthetic pigment contents (mg·g^−1^) using an ultraviolet spectrophotometer (model. T-6, Nanjing Feile Instrument Co., Ltd., Nanjing, China). The calculation formula was described in a previous paper [82].

### 4.3. Detection of Sugar Contents

The fresh needles were harvested, immediately frozen in liquid nitrogen, and stored at −80 °C until the analysis. The sample was crushed using a mixer mill with a zirconia bead for 1.5 min at 30 Hz after freeze-drying. 20 mg of powder was diluted to a volume of 500 μL with methanol/isopropanol/water (3:3:2, *v*/*v*/*v*), vortexed for 3 min, and ultrasound for 30 min. The extract was centrifuged at 14,000 rpm for 3 min at 4 °C. 50 μL of the supernatant was mixed with internal standard and evaporated under a nitrogen gas stream. The evaporated sample was transferred to the lyophilizer for freeze-drying. The residue was used for further derivatization. The derivatization method was as follows: the sample of small molecular carbohydrates was mixed with a 100 μL solution of methoxyamine hydrochloride in pyridine (15 mg/mL). The mixture was incubated at 37 °C for 2 h. Then, 100 μL of BSTFA was added to the mixture and kept at 37 °C for 30 min after vortex-mixing. The mixture was analyzed by GC–MS/MS after diluting to an appropriate concentration. Sugar contents were detected by MetWare (Wuhan, China) based on the Agilent 7890B-7000D platform.

### 4.4. Detection of Hormone Contents

The fresh needles were harvested, immediately frozen in liquid nitrogen, and stored at −80 °C until the analysis. The sample (50 mg fresh weight) was ground into powder under liquid nitrogen and extracted with 1 mL of methanol/water/formic acid (15:4:1, *v/v/v*). The combined solution was evaporated to dryness under a nitrogen gas stream, and the dried extract was reconstituted in 100 µL of 80% methanol (*v*/*v*). Subsequently, the reaction solution was filtered with a 0.22 μm filter for the subsequent LC-MS analysis. The contents of jasmonates (JAs), salicylic acids (SAs), and gibberellins (GAs) were detected by MetWare (Wuhan, China) based on the AB Sciex QTRAP 6500 LC-MS/MS platform.

### 4.5. PacBio Iso-Seq Library Construction, Sequencing, and Gene Functional Annotation

Total RNA was isolated from each sample with the polysaccharide polyphenol plant total RNA extraction kit (Tiangen, Beijing, China). The quality and quantity of total RNA were determined using a 2100 Bioanalyzer (Agilent Technologies, Santa Clara, CA, USA) and ND-2000 (NanoDrop Thermo Scientific, Wilmington, DE, USA), respectively. Total RNA from six independent samples (high-yield and common-yield with three biological replicates) was mixed at equal ratios. One mixed Iso-Seq library was constructed according to the Isoform Sequencing protocol (Iso-Seq) using the Clontech SMARTer PCR cDNA Synthesis Kit and the BluePippin Size Selection System protocol as described by Pacific Biosciences (PN 100-092-800-03). Sequence data of PacBio Sequel was processed using the SMRTlink V7.0 software (https://www.pacb.com/support/software-downloads/ (accessed on 6 December 2019)). Circular consensus sequences (CCSs) were generated from subread BAM files by setting the parameters to min_length 50, max_drop_fraction 0.8, no_polish TRUE, min_zscore -9999.0, min_passes 1, min_predicted_accuracy 0.8, max_length 15,000. CCS.BAM files were output, which were then classified into full-length and non-full-length reads using lima, removing polyA by refinement. Full-length fasta files were fed into the cluster step, corrected by isoform-level clustering (ICE), and then final arrow polishing was conducted, parameters setting as: hq_quiver_min_accuracy 0.99, bin_by_primer false, bin_size_kb 1, qv_trim_5p 100, qv_trim_3p 30. Additional nucleotide errors in consensus reads were corrected using LoRDEC software (http://atgc.lirmm.fr/lordec (accessed on 15 December 2019)) based on the Illumina RNA-Seq data. The redundancy in corrected consensus reads was removed by CD-HIT software (https://github.com/weizhongli/cdhit, -c 0.95 -T 6 -G 0 -aL 0.00 -aS 0.99 -AS 30 (accessed on 28 December 2019)) to obtain final transcripts for the further analysis.

Gene function was annotated based on the following databases: NCBI Non-redundant Protein (Nr, cut-off E value ≤ 1 × 10^−10^), NCBI Non-redundant Nucleotide (Nt, cut-off E value ≤ 1 × 10^−10^), protein family (Pfam, cut-off E-value ≤ 0.01), euKaryotic Ortholog Groups (KOG, cut-off E value ≤ 1 × 10^−10^), Swiss-Prot protein (cut-off E value ≤ 1 × 10^−10^), Kyoto Encyclopedia of Genes and Genomes (KEGG, cut-off E value ≤ 1 × 10^−10^), and Gene Ontology (GO, cut-off E value ≤ 1 × 10^−10^). Protein coding sequences from cDNA were identified using the ANGEL pipeline (a long-read implementation of ANGLE). The closely related species were used to ensure that the protein sequences were ANGEL-trained and then performed the ANGEL prediction for given sequences.

### 4.6. RNA-Seq Library Construction, Sequencing, and Analysis of Differentially Expressed Genes

All 6 qualified total RNA from six independent samples (high-yield and common-yield with three biological replicates) were used to construct a transcriptome sequencing library. The transcriptome library was pair-end sequenced on the Illumina HiSeq Xten platform (Illumina Inc., San Diego, CA, USA). The clean reads obtained were used for error correction to generate the polished consensus sequences as described above. Gene expression levels were estimated by RSEM [83] for each sample. The relative expression levels were normalized by FPKM (fragments per kilobase of transcript sequence per millions of mapped reads). Differential expressed genes (DEGs) were identified by DESeq [84] setting an adjusted *p*-value < 0.05 and |log2(fold change)| ≥ 1. GOseq R package (http://www.bioconductor.org/packages/release/bioc/html/goseq.html (accessed on 10 February 2020)) and KOBAS software (http://kobas.cbi.pku.edu.cn/download.php (accessed on 18 March 2020)) were used to perform GO and KEGG enrichment analyses on DEGs, respectively.

### 4.7. Protein Extraction, iTRAQ Labeling, and LC-MS/MS Analysis

The sample was ground in liquid nitrogen, then the powder was transferred to 5 mL centrifuge tubes, and sonicated three times on ice in lysis buffer (50 mmol/L EDTA, 700 mmol/L sucrose, 100 mmol/L KCl, 500 mmol/L Tris-HCl, pH 8.0). An equal volume of Tris-saturated phenol (pH 8.0) was added, then the mixture was vortexed for 5 min, and centrifuged at 4 °C, 5000× *g* for 10 min. The supernatant was removed, mixed with a 4× volume of chilled methanol containing 0.1 mol/L ammonium acetate, and incubated at −20 °C overnight. After centrifugation at 4 °C, 5000× *g*, for 10 min, the supernatant was discarded. The precipitate was washed with ice-cold methanol once, followed by ice-cold acetone three times. The remaining pellet was then air-dried, and dissolved in lysis buffer (7 M urea, 2 M thiourea, 20 mM Tris-HCl, pH 8.0–8.5), and the protein concentration was detected by the Bradford method. For digestion, the protein solution was reduced with 10 μL reducing reagent for 1 h at 37 °C and alkylated with 2 μL cysteine-blocking reagent for 30 min at room temperature. After digestion with trypsin, the peptides were dried by vacuum freeze centrifugation and reconstituted in triethylammonium bicarbonate buffer (TEAB). The digested peptides were labeled with iTRAQ reagents (Applied Biosystems, Foster City, CA, USA) according to the manufacturer’s instructions. Common-yield samples were labeled iTRAQ tags 113, 114, and 115, and high-yield samples were labeled with iTRAQ tags 116, 119, and 121, respectively. Equal amounts of iTRAQ-labelled peptides were mixed, dried, then reconstituted in buffer A (2% acetonitrile, 98% water with ammonia at pH 10), and fractionated by HPLC (RIGOL, Beijing, China). Then, the peptides were redissolved with 0.1% formic acid (FA) and analyzed by a Q-Exactive HF mass spectrometer (Thermo Fisher Scientific, Waltham, MA, USA) coupled with a nano-HPLC instrument (UltiMate 3000 LC system, Dionex, CA, USA). The mass spectrometer (MS) was operated in the data-dependent mode with positive polarity at an electrospray voltage of 2.0 kV. The m/z scan range was 300 to 1400 for the full scan, and the top 20 intense ions were detected to use for MS/MS.

### 4.8. Protein Identification and Functional Annotation

The MS raw data was analyzed with Proteome Discoverer V1.4 software (Thermo Fisher Scientific, Waltham, MA, USA) using the Mascot search engine to search against the masson pine database, including 17,323 sequences from Iso-Seq constructed as described above. The search parameters were set as follows: precursor mass tolerance was 15 p.p.m., fragment tolerance was 20 m.m.u., the dynamic modifications were oxidation (M) and iTRAQ labeling (K, Y, and N-term), the static modification was carbamidomethyl (C), and up to two missed cleavages were allowed. Proteins with only fold change > 1.2 (the average of all comparison group ratios) and false discovery rate (FDR) < 0.05 were defined as differentially expressed proteins (DEPs). All proteins identified were functionally annotated and classified by GO, KOG, and KEGG databases. DEPs were analyzed using the GO and KEGG databases to identify significantly enriched functional categories and metabolic pathways.

### 4.9. Quantitative Real-Time PCR Assay

A quantitative real-time PCR assay (qRT-PCR) was used to validate the expression profiles of the candidate genes. Total RNA samples were the same samples used for the cDNA library preparation. RNA was extracted as described for cDNA library construction, and cDNA was reversely transcribed using a PrimeScript RT reagent kit containing gDNA Eraser (Takara, Dalian, China). Primers were designed with Primer Premier 5.0 software (Primier Biosof international., Quebec, QC, Canada). The primers designed were synthesized in Sangon Biotech (Shanghai) Co., Ltd. qRT-PCR was constructed using Maxima^®^ SYBR Green/ROX qPCR Master Mix (2X) (Thermo Fisher Scientific, Waltham, MA, USA) on CFX96 Real-Time PCR Manager 3.1 (BioRad, Hercules, CA, USA). A 10 μL reaction containing 1 μL synthesized cDNA, 5 μL 2-TB Green Premix Ex Taq mix, 0.5 μL 10 mM forward primer, 0.3 μL 10 mM reverse primer, and 3 μL sterile distilled water was amplified. PCR amplification was performed as follows: preheating at 94 °C for 10 min, followed by 40 cycles at 94 °C for 15 s, 60 °C for 30 s, 72 °C for 30 s, and the melting curves were generated from 65 °C to 95 °C with increments of 0.5 °C every 5 s. The reference gene SKI-interacting protein was applied as the internal control [38], and the primers used in this study are listed in Table S1. Each sample was performed with three biological replicates and three technical replicates. The relative expression levels of genes were computed by the 2^−ΔΔCt^ method [85].

### 4.10. Statistical Analysis

The biochemical characteristics and qRT-PCR data from high and common resin yield samples were performed by SPSS 18.0 software (SPSS, Chicago, IL, USA). Analyses of variance (ANOVA) for sets of data were analyzed using Duncan’s testing method to determine differences between pairs of means of multiple experiments. *p* < 0.05 and *p* < 0.01 were considered to be significant and extremely significant, respectively. The relationships between the expression levels of DEPs and their corresponding mRNAs were assessed using the linear least-squares, and correlation coefficients were calculated with the Pearson correlation test.

## 5. Conclusions

In the present study, the differences in biochemical indexes of photosynthetic pigment, sugar, and hormone indicated great variations in photosynthesis, carbohydrate metabolism, and hormone biosynthesis between different resin yields, which may be related to the differences in resin yield. Subsequently, an integrated transcriptomic and proteomic analysis of masson pine with high and common resin yield was conducted, and some important metabolic pathways were revealed, such as terpenoid biosynthesis, photosynthesis, carbohydrate metabolism, and hormone biosynthesis. Furthermore, several key genes referred to as terpenoid biosynthesis showed the same trend at the transcriptional and proteomic levels, including *MonoTPS1*, *SesquiTPS1*, *SesquiTPS2*, *SesquiTPS3*, *PMK*, and *MVD*, suggesting that they were closely correlated with resin yield. Finally, the integrative analysis of the transcriptome, proteome, and biochemical characteristics revealed some important candidate genes that were highly related to resin yield, including *chlG*, *HXK*, *SUS*, *PGK*, *PDH*, *DLST*, *OPR*, and *JMT*. In conclusion, terpenoid biosynthesis, carbohydrate metabolism, photosynthesis, and hormone biosynthesis may play crucial roles in the regulation of resin yield, and some key genes involved in these pathways may be candidates that influence the resin yield. These findings not only expand the understanding of the molecular regulatory mechanisms of resin yield but also provide candidate genes that can be applied to facilitate genetic improvement of high resin yield in masson pine.

## Figures and Tables

**Table 1:** Summary of PacBio transcripts of masson pine needles in the high and common resin yield.

	PacBio	
	Before Correct	After Correct
Subreads base (G)	23.92	
Subreads number	15,216,963	
Average subreads length	1572	
N50 (subreads)	1893	
CCS_number	520,718	
Full length	446,770	
FLNC	416,262	
Average FLNC read length	1719	
Consensus reads	38,784	
Total_nucleotides	72,040,170	72,013,662
Total_number	38,784	38,784
Mean_length	1858	1857
Min_length	75	75
Max_length	9057	9060
N50 (consensus)	2143	2142
N90 (consensus)	1139	1138
Number of transcripts		38,784
Number of Genes		17,266
Number of genes annotated		98.30%
Number of genes unannotated		1.70%

## Data Availability

Not applicable.

## Supplementary Materials

The supporting information can be downloaded at: https://www.mdpi.com/article/10.3390/ijms231911420/s1.
