# Supplementary material for: Identification of Genes and Metabolic Pathways Involved in Resin Yield in Masson Pine by Integrative Analysis of Transcriptome, Proteome and Biochemical Characteristics
Source: Int J Mol Sci. 2022 Sep 28;23(19):11420. doi: 10.3390/ijms231911420 (PMC9570031; doi:10.3390/ijms231911420)
Supplement: Supplementary file 1 [file ijms-23-11420-s001.zip › Supplementary Figures.pdf]

Supplementary Figures

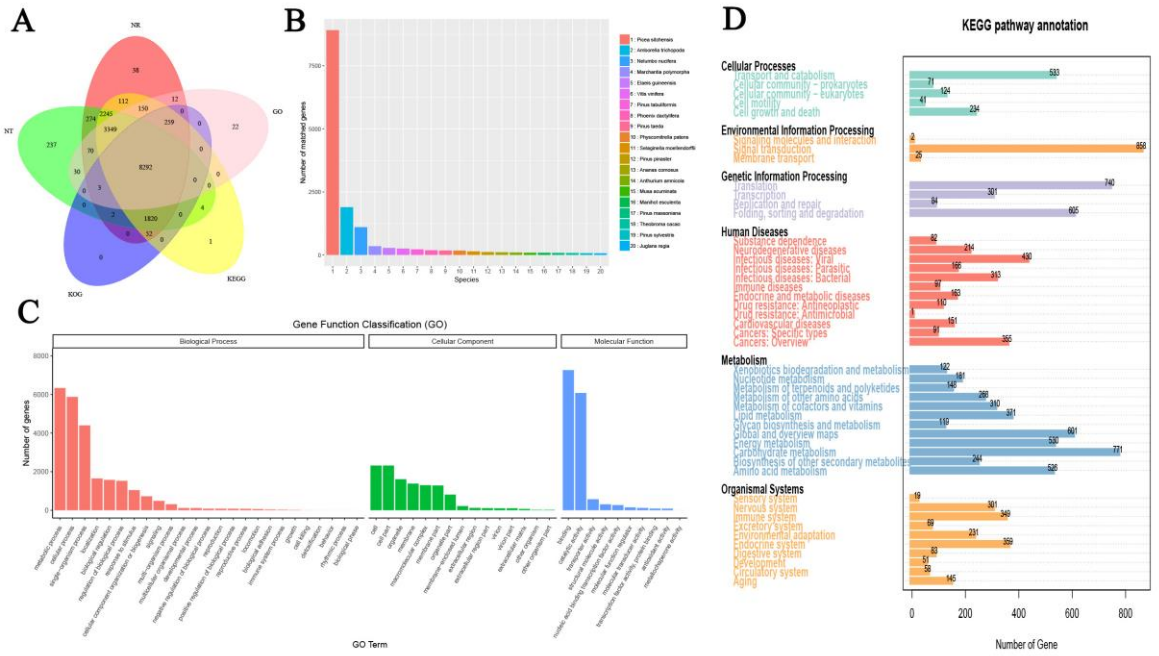

Figure S1. Annotation of masson pine transcripts. (A) Gene functional annotations in 5 databases (Nr, Nt, GO, KEGG, KOG). (B) Homologous species distribution of masson pine annotated in the Nr database. (C) Annotation of the GO function of masson pine transcripts. (D) Annotation of the KEGG function of masson pine transcripts.

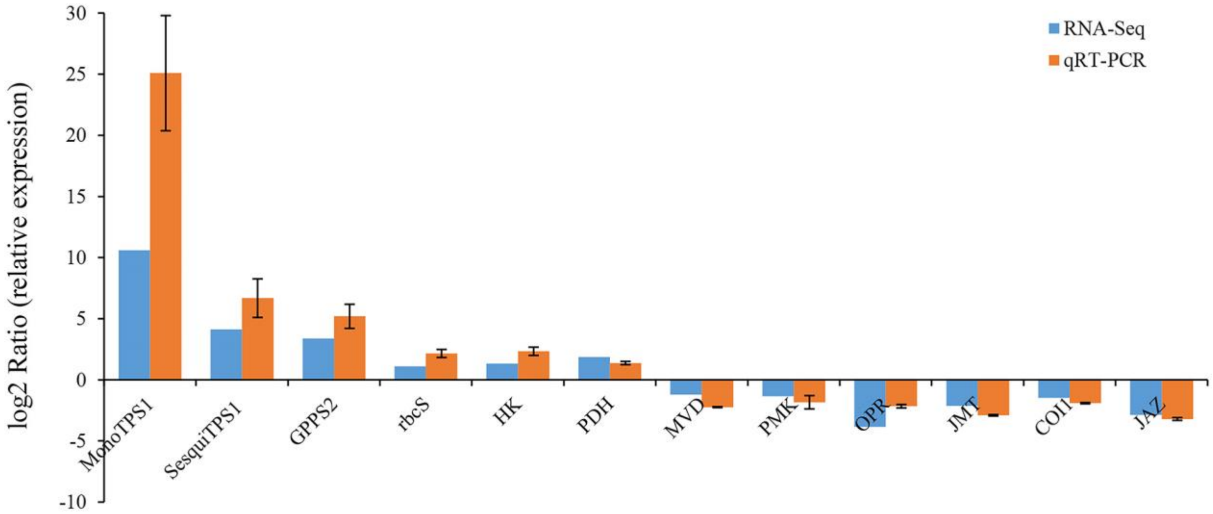

Figure S2. qRT-PCR validated the expression of the key genes involved in the resin yield. The blue and orange bars represent the relative gene expression levels calculated by RNA-Seq and qRT-PCR, respectively. The relative gene expression levels were calculated by $2^{-\Delta\Delta CT}$ using the reference gene *SKI*. Data were shown as the means ± SE (n=3).

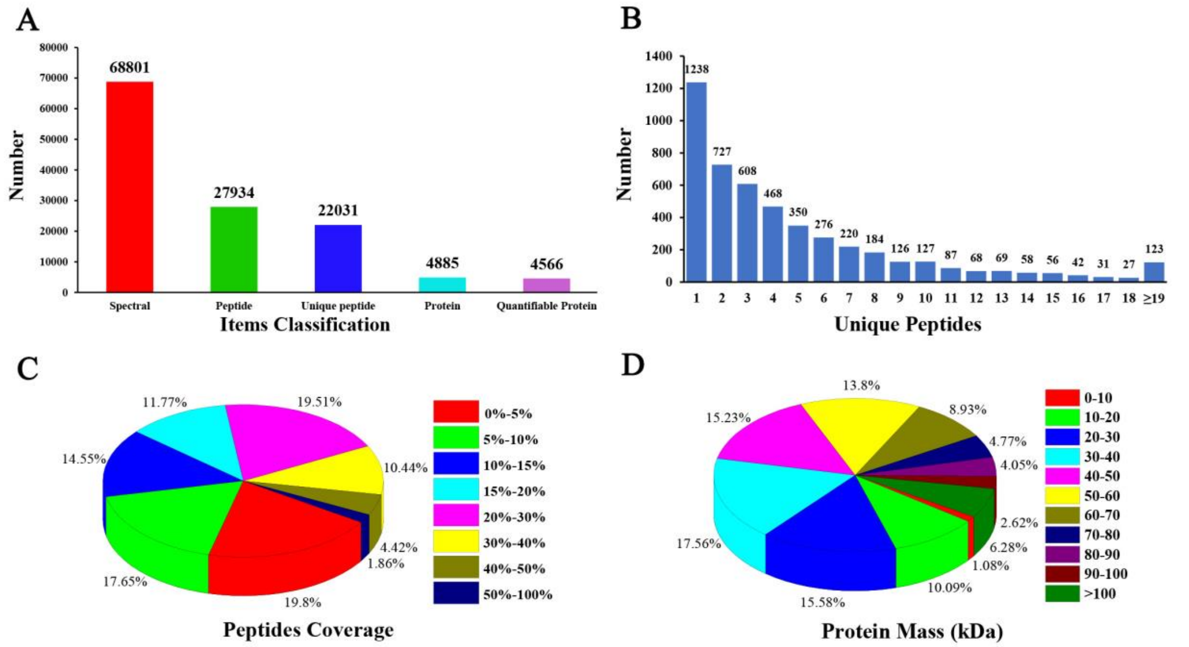

Figure S3. Protein species identification of the isobaric tags for the relative and absolute quantitation (iTRAQ) analysis in masson pine needles. (A) Number of spectra, peptide and protein. (B) Number of unique peptides that were matched to proteins. The X-axis shows the unique peptide number of each protein, and the Y-axis shows the corresponding protein number. (C) Distribution of peptide coverage ranges of identified proteins. (D) Distribution of molecular mass of identified proteins.

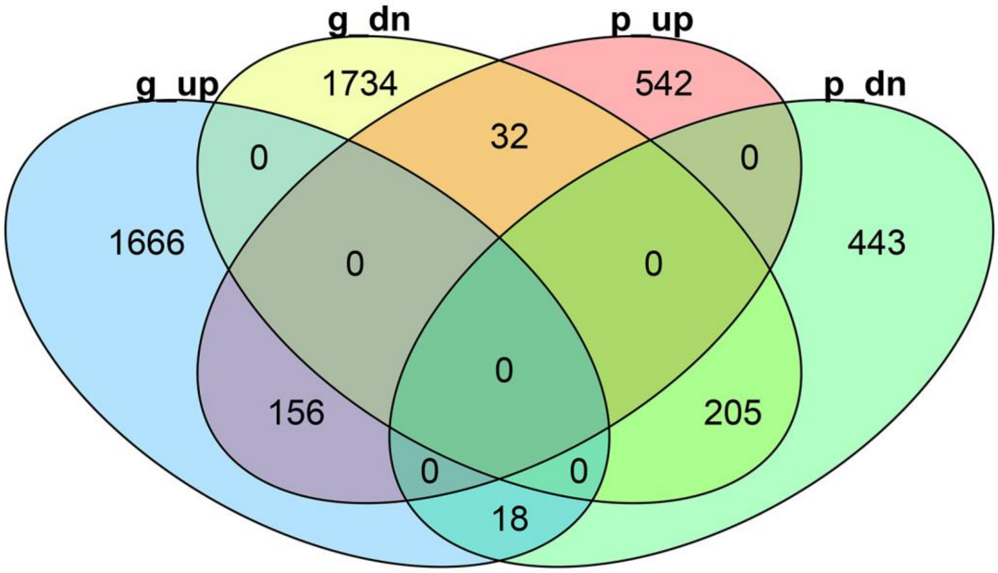

Figure S4. Venn diagram for comparative analysis of DEGs/DEPs. DEGs/DEPs were divided into two groups of up-regulated or down-regulated, respectively. Each group was indicated by expression level (“g” for gene and “p” for protein, respectively) and regulatory direction (“up” for up-regulated and “dn” for down-regulated, respectively).
